# Confidence-guided integration of otoscopy videos and tympanometry for middle ear disease diagnosis: A multi-center otoscopy study with external paired-cohort evaluation

**DOI:** 10.1371/journal.pone.0357492

**Published:** 2026-09-01

**Authors:** Hao Lu, Carl D. Langefeld, Amy Zinnia, Muhammet Fatih Demir, Shalaka Chavan, Charles A. Elmaraghy, Margaret Wilson, Muhammad Khalid Khan Niazi, Aaron C. Moberly, Metin N. Gurcan

**Affiliations:** 1 Center for Artificial Intelligence Research, Wake Forest University School of Medicine, Winston-Salem, North Carolina, United States of America; 2 Department of Biostatistics and Data Science, Wake Forest University School of Medicine, Winston-Salem, North Carolina, United States of America; 3 Department of Otolaryngology—Head and Neck Surgery, Nationwide Children’s Hospital and The Ohio State University, Columbus, Ohio, United States of America; 4 Department of Otolaryngology-Head and Neck Surgery, Vanderbilt University Medical Center, Nashville, Tennessee, United States of America; 5 Department of Pathology, The Ohio State University, Columbus, Ohio, United States of America; LSU Health Shreveport, UNITED STATES OF AMERICA

## Abstract

Accurate diagnosis of middle ear diseases using otoscopy remains challenging, particularly in primary care settings where clinician experience with otoscopy varies and visual examination alone may not fully capture middle ear physiology. Although AI-based models have shown promise for automated otoscopy interpretation, most rely primarily on visual information, creating an opportunity to improve diagnostic robustness by integrating complementary physiological measurements. Here, we present OtoTymp-AI, a confidence-guided multimodal decision-fusion framework that integrates otoscopy video analysis with conventional 226 Hz tympanometry. A convolutional neural network was trained to classify otoscopy videos, and tympanometric measurements were incorporated through a clinically interpretable, rule-guided decision strategy to refine visually uncertain predictions rather than through jointly trained multimodal representation learning. Within this multi-center otoscopy study, multimodal evaluation was performed in one independent external cohort comprising 104 videos with paired tympanometry across six diagnostic categories. In this exploratory paired-cohort analysis, the video-only CNN achieved an overall accuracy of 63.46% (95% CI, 53.88–72.08), while the hybrid video-plus-tympanometry framework achieved 81.73% accuracy (95% CI, 73.22–87.98) at the best observed threshold of 0.90, corresponding to an observed absolute improvement of 18.27 percentage points over the CNN-only model (95% paired bootstrap CI, 9.62–26.92). Improvements were observed for selected categories represented in this cohort, including effusion and retraction, although confidence intervals were wide for categories with small positive sample sizes. These findings suggest that integrating anatomical video information with physiological tympanometry may improve AI-assisted middle ear diagnosis in this external paired cohort, but the operating threshold and per-class performance require confirmation in larger prospective paired multimodal cohorts.

## Introduction

Otoscopy is a fundamental, routinely performed examination for evaluating the external auditory canal, tympanic membrane, and middle ear. Ear-related complaints account for a substantial proportion of visits in primary care and pediatric settings, where clinicians are frequently required to assess ear pathology during both routine and acute care encounters [1–3]. However, access to otolaryngology specialists remains limited due to workforce shortages and long wait times, resulting in most initial evaluations being performed by non-specialist clinicians [2,4,5].

Despite its central role in the evaluation of ear disease, the diagnostic accuracy of clinicians performing otoscopy in primary care settings varies widely and is strongly influenced by clinician training and experience [6,7]. Visual interpretation of the tympanic membrane can be challenging, particularly in cases with subtle or overlapping features. Inaccurate diagnosis may lead to inappropriate treatment, delayed care, and adverse outcomes including hearing loss and reduced quality of life [8].

Recent advances in artificial intelligence (AI) have enabled the development of deep learning models for automated interpretation of otoscopic images and videos [9–12]. These models have demonstrated promising performance in controlled settings. However, most existing AI-based otoscopy models rely primarily on visual information and are often developed using single-center datasets [13], creating an opportunity to improve diagnostic robustness through external validation and integration of complementary clinical measurements. In particular, visual assessment alone may not fully distinguish conditions with similar appearance but different underlying middle-ear physiology.

In clinical practice, otoscopy is often complemented by tympanometry [14–16], a non-invasive test that evaluates the mechanical properties of the tympanic membrane and middle ear system. Tympanometry provides objective physiological information that can help differentiate between conditions that are visually ambiguous, such as distinguishing effusion from a normal tympanic membrane. This complementarity creates an opportunity for AI-based diagnostic frameworks that integrate anatomical information from otoscopy with physiological information from tympanometry.

In this study, we propose OtoTymp-AI, a confidence-guided multimodal decision-fusion framework that combines otoscopy video analysis with tympanometric measurements. Rather than learning a joint multimodal representation, OtoTymp-AI uses a clinically interpretable, confidence-triggered decision strategy: high-confidence video predictions are preserved, whereas visually uncertain predictions are refined using physiologically grounded tympanometry rules. This design was intended to evaluate whether complementary physiological information can improve AI-assisted middle ear diagnosis in a setting where paired otoscopy–tympanometry data are difficult to collect.

To evaluate this framework, we conducted a retrospective multi-center study using otoscopy videos from three independent institutions. The Nationwide Children’s Hospital (NCH)-training dataset was used for model development, The Ohio State University (OSU)-validation dataset was used for model selection, and the Vanderbilt University Medical Center (VUMC)-external test dataset provided paired otoscopy videos and conventional 226 Hz tympanometry for multimodal evaluation. OtoTymp-AI was evaluated across six clinically relevant categories: effusion, normal, perforation, retraction, tympanostomy tube presence, and tympanosclerosis. This study demonstrates the potential of combining anatomical video information with physiological testing to improve AI-assisted middle ear diagnosis in an independent external clinical cohort. Accordingly, the multi-center design applies to otoscopy video model development, validation, and external testing, whereas the video-plus-tympanometry multimodal evaluation was performed in the single VUMC external paired cohort where ear-specific tympanometry was available.

## Related work

### AI-based interpretation of otoscopy images

Early applications of AI in otology have primarily focused on static otoscopic images. Convolutional neural networks (CNNs) have been widely used to classify tympanic membrane conditions, with several studies [9–11,17] reporting high diagnostic accuracy under controlled conditions. These approaches typically rely on curated image datasets with well-centered views of the tympanic membrane and limited variability in acquisition settings.

Despite promising results, static image–based methods have several practical limitations. Even a carefully selected single frame may not capture the entire tympanic membrane, particularly in pediatric otoscopy, where the narrower external auditory canal can restrict the field of view. As a result, diagnostically relevant findings may be visible only across multiple complementary views rather than in one image alone. In addition, many image datasets are collected from a single institution or under standardized acquisition conditions, raising concerns about generalizability to real-world clinical environments. Static images may also represent idealized views rather than the full variability encountered during routine otoscopy. Therefore, while static image models provide an important foundation, video-based analysis offers an opportunity to use multiple selected frames for more comprehensive diagnostic assessment.

### Video-based otoscopy analysis

To address the limitations of static image analysis, more recent studies [18–20] have explored video-based approaches for otoscopic diagnosis. These methods either aggregate information across multiple frames or select diagnostically informative frames from video sequences to generate video-level predictions. By incorporating multiple viewpoints over time, video-based models can provide more complete coverage of the tympanic membrane—an advantage that is particularly important in pediatric otoscopy, where the restricted field of view often prevents a single image from capturing the entire eardrum. Video-based models can also better handle variability in image quality and reflect the dynamic nature of clinical examinations, making them more suitable for real-world deployment.

However, several challenges remain for video-based otoscopy analysis. Existing approaches often focus on visual information alone, and performance is frequently evaluated using internal or randomly split datasets, with limited validation on independent external cohorts [21]. As a result, the robustness of video-based AI models across institutions, devices, and patient populations remains an important area for further study. In addition, visual information alone may not fully distinguish conditions with similar otoscopic appearance but different underlying middle-ear physiology.

### Multimodal approaches and tympanometry

Tympanometry is a well-established clinical tool that provides objective assessment of tympanic membrane and middle ear function by measuring the mechanical properties of the tympanic membrane and middle ear system [22]. It is routinely used in conjunction with otoscopy to improve diagnostic accuracy, particularly in differentiating conditions such as middle ear effusion, perforation, and normal ear status [23].

Despite its clinical importance, tympanometry is not commonly incorporated into AI-based otoscopy frameworks. Prior work has explored decision fusion between image analysis and tympanometry [14], but multimodal AI approaches that combine otoscopy videos with tympanometric measurements remain limited. This represents an important opportunity because the two modalities provide complementary information: otoscopy captures anatomical appearance, whereas tympanometry reflects middle-ear mechanical function. Integrating these modalities may help refine visually uncertain predictions, particularly for conditions with overlapping otoscopic features but distinct physiological patterns.

Together, these observations motivate an interpretable decision-fusion approach that uses tympanometry-derived physiological information to refine uncertain video-based AI predictions. This formulation targets transparent clinical decision support and is distinct from jointly trained multimodal representation learning.

## Materials and methods

### Data

**Fig 1:**
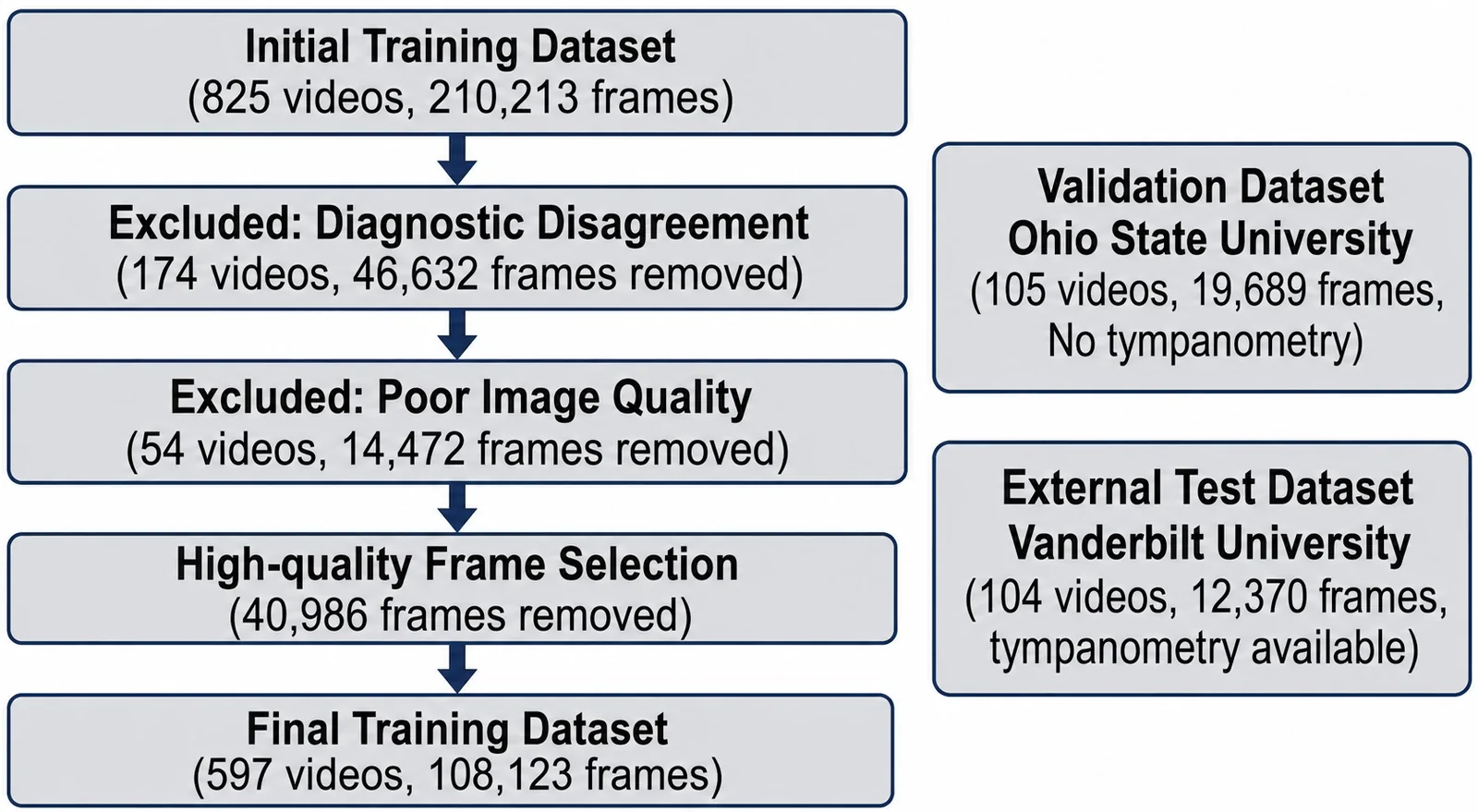
Dataset construction pipeline and cohort distribution across training, validation, and external test sets. Otoscopy videos were collected from three independent institutions and assigned to patient-disjoint cohorts. The NCH-training dataset underwent diagnostic agreement screening, video-level quality filtering, and automated high-quality frame selection for model development. The OSU-validation dataset was used for model selection without additional case-level exclusion, and the VUMC-external test dataset retained all otoscopy videos with available paired conventional 226 Hz tympanometry for multimodal evaluation. The final analysis cohort included 806 otoscopy videos: 597 training videos, 105 validation videos, and 104 external test videos.

To evaluate model generalizability and prevent data leakage, the training, validation, and test datasets were strictly separated at both the institutional and patient levels. The NCH-training dataset was collected from Nationwide Children’s Hospital and used for model development. The OSU-validation dataset was collected from The Ohio State University and used for model selection. The VUMC-external test dataset was collected from Vanderbilt University Medical Center and included paired conventional 226 Hz tympanometry measurements collected as an additional research-related follow-up examination after the otoscopy video diagnosis had been completed. Tympanometry measurements were matched to the corresponding ear examination, enabling multimodal evaluation while preserving independence between the video-based reference diagnosis and tympanometry-derived model input. The cohort construction and institutional split are illustrated in Fig 1, and the final analysis cohorts are summarized in Tables 1 and 2. After quality control and high-quality frame selection, the final analysis cohort included 806 otoscopy videos from three institutions: 597 videos in the NCH-training dataset, 105 videos in the OSU-validation dataset, and 104 videos in the VUMC-external test dataset.

**Table 1:** Final dataset summary after quality control and high-quality frame selection.

Dataset Split	Institution	Videos	Frames	Tympanometry Available
Training	Nationwide Children’s Hospital	597	108,123	No
Validation	OSU	105	19,689	No
External Test	VUMC	104	12,370	Yes

**Table 2:** Distribution of videos and selected frames by diagnostic category across the NCH-training, OSU-validation, and VUMC-external test datasets.

Diagnosis	Training Frames	Training Videos	Validation Frames	Validation Videos	Test Frames	Test Videos
AOM	11,212 (10.37%)	50 (8.38%)	2,471 (12.55%)	15 (14.29%)	0 (0.00%)	0 (0.00%)
Effusion	24,135 (22.32%)	107 (17.92%)	4,377 (22.23%)	21 (20.00%)	1,541 (12.46%)	11 (10.57%)
Normal	27,177 (25.14%)	187 (31.32%)	3,835 (19.48%)	30 (28.57%)	2,401 (19.41%)	35 (33.65%)
Perforation	16,860 (15.59%)	85 (14.24%)	4,106 (20.85%)	13 (12.38%)	2,640 (21.34%)	20 (19.23%)
Retraction	11,127 (10.29%)	54 (9.05%)	2,517 (12.78%)	10 (9.52%)	2,838 (22.94%)	20 (19.23%)
Tympanostomy Tube	8,133 (7.52%)	65 (10.89%)	987 (5.01%)	9 (8.57%)	1,643 (13.28%)	10 (9.61%)
Tympanosclerosis	9,479 (8.77%)	49 (8.21%)	1,396 (7.09%)	7 (6.67%)	1,307 (10.57%)	8 (7.69%)
**Total**	**108,123**	**597**	**19,689**	**105**	**12,370**	**104**

Reference standard labels were assigned as described in the “Reference Standard and Blinding” section below. Briefly, otoscopy videos were independently reviewed by at least two experienced clinicians, and the reference diagnosis was determined from otoscopic/video findings without use of tympanometry results. Cases with initial diagnostic disagreement were reviewed through consensus discussion; videos for which a consensus primary diagnosis could not be reached were excluded. Videos were also excluded if visualization of the tympanic membrane was severely compromised by motion artifacts, poor image quality, or obstruction by cerumen or debris, as determined through clinician review.

The NCH-training dataset underwent training-cohort curation, including diagnostic agreement screening and video-level quality filtering, before automated high-quality frame selection. In contrast, the OSU-validation dataset was used without additional case-level exclusion, and the VUMC external paired cohort retained all otoscopy videos with available matched tympanometry. Automated high-quality frame selection was applied to videos for frame-level input preparation, but it did not remove additional OSU-validation or VUMC-external test cases from the analysis cohort. The primary analysis was performed at the ear-examination level. To maintain patient-disjoint dataset splits, all examinations from the same patient were assigned to only one dataset split; therefore, no patient contributed ear examinations to more than one split. Because some patients contributed bilateral ear examinations within the same split, we also summarized the number of unique patients and bilateral-ear contributors and performed patient-level sensitivity analyses to evaluate whether the main findings were sensitive to within-patient correlation. Fig 1 illustrates dataset curation and multi-institutional cohort composition.

For the external test dataset, tympanometry measurements were paired with otoscopy videos after the clinician-assigned video reference diagnoses had been finalized. Matching between modalities was performed using study and clinical records to ensure correspondence to the same patient ear. Because tympanometry was collected as an additional research-related follow-up examination and required ear-specific matching, paired tympanometry was available only for a subset of the external cohort, contributing to the modest size of the paired multimodal test set. In the VUMC external paired cohort, the final analysis included 104 ear-level examinations from 76 unique patients. Twenty-eight patients contributed bilateral ear examinations, accounting for 56 ear-level examinations, whereas 48 patients contributed one ear examination. The cohort included 56 right-ear and 48 left-ear examinations. Although AOM cases were present in the NCH-training and OSU-validation datasets, no AOM cases were available in the VUMC external paired cohort after cohort curation. Therefore, external paired-cohort performance estimates were not calculated for AOM, and conclusions regarding multimodal performance should not be extended to AOM.

The VUMC external paired cohort included 104 adult ear-level examinations. The median age was 51.0 years (IQR, 33.0–65.0; range, 18–85), and no pediatric cases were included. By age group, 43 examinations were from patients aged 18–44 years, 33 from patients aged 45–64 years, and 28 from patients aged ≥65 years.

### Reference standard and blinding

The reference standard for model training and evaluation was defined at the ear-examination/video level using clinician-assigned primary diagnoses based only on otoscopic video review. Each otoscopy video was independently reviewed by at least two experienced clinicians. During reference-label assignment, reviewers had access to the otoscopy video only and did not have access to tympanometry results, model predictions, or the hybrid model output. Reviewers assigned a primary diagnosis based on visual otoscopic findings observed in the video, including tympanic membrane appearance, position, opacity, structural abnormalities, and visible evidence of tympanostomy tube placement or tympanosclerosis.

Tympanometry results were not used to assign the reference standard labels. For the VUMC external paired cohort, expert video diagnoses were completed before tympanometry data were collected or made available for this study. Conventional 226 Hz tympanometry was collected subsequently as an additional research-related follow-up examination and was paired with the corresponding otoscopy video only after the reference labels had been finalized. Tympanometry measurements were used solely as an input to the rule-guided hybrid decision-fusion model and did not contribute to the reference standard. Therefore, the same tympanometry measurements used by the hybrid model were not used by the annotators and did not create incorporation bias in the reference diagnosis.

Clinicians assigning the reference labels were blinded to tympanometry results and model predictions. For cases with an initial disagreement between reviewers, the case was discussed by the reviewers to determine whether a consensus primary diagnosis could be reached. If consensus could be reached, the consensus diagnosis was used as the reference label. If no consensus diagnosis could be established, the video was excluded from the final analysis cohort. For the VUMC external paired test cohort, no videos were excluded because of diagnostic disagreement, poor visualization, or video-level quality concerns after paired-cohort assignment; all 104 videos with available matched tympanometry were retained for analysis. In cases with multiple coexisting otologic findings, a single primary diagnosis was assigned according to the clinically dominant finding for the purposes of single-label model evaluation; secondary findings were not used as additional ground-truth labels.

### Ethics statement

This retrospective, multi-center study was approved by the Wake Forest University School of Medicine Institutional Review Board under approval number IRB00048334. The requirement for informed consent was waived by the IRB because the study used retrospective, de-identified clinical data. Data were accessed for research purposes between January 10, 2026 and March 15, 2026. All otoscopy videos, tympanometry records, and associated clinical labels were de-identified before analysis. The authors did not have access to information that could identify individual participants during or after data collection.

### Diagnostic framework

#### Overview of the diagnostic framework.

**Fig 2:**
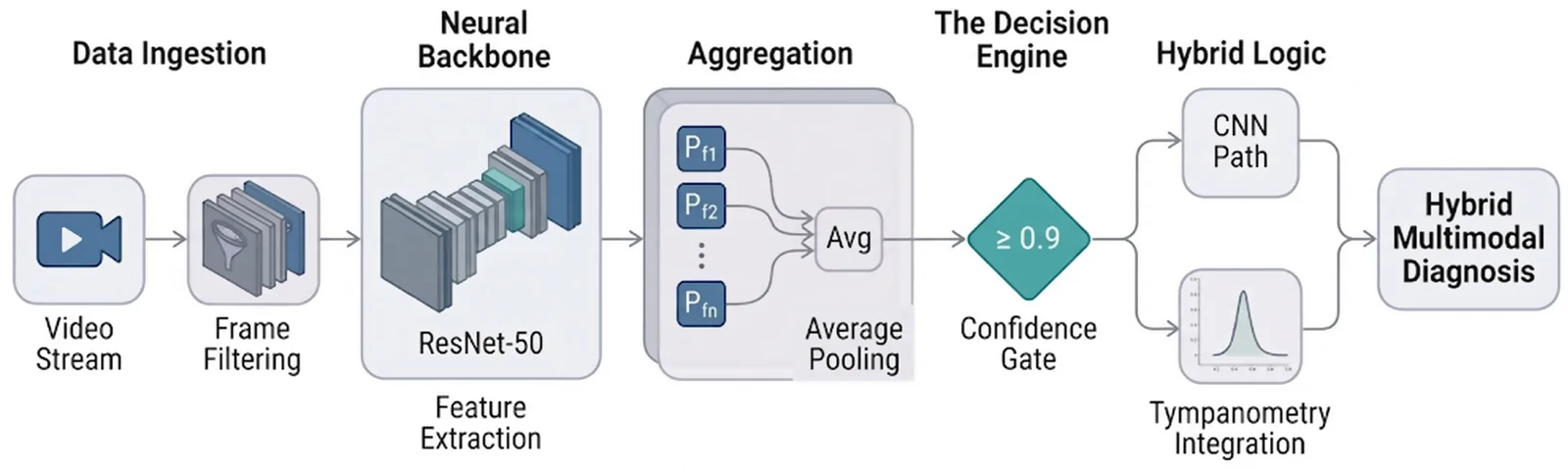
Overview of OtoTymp-AI. The framework consists of four components: automated high-quality frame selection, training-time data augmentation, video-level CNN classification using averaged frame-level probabilities, and confidence-guided tympanometry refinement. High-confidence video predictions are retained as the final AI diagnosis, whereas visually uncertain predictions are refined using clinically interpretable tympanometry rules. This design combines anatomical information from otoscopy videos with physiological information from tympanometry.

**OtoTymp-AI** consists of four main components: (1) automated high-quality frame selection from otoscopy videos, (2) training-time data augmentation to improve cross-center robustness, (3) video-level AI classification by aggregating frame-level CNN predictions, and (4) confidence-guided multimodal refinement using tympanometry measurements in visually uncertain cases. An overview of the pipeline is illustrated in Fig 2.

#### High-quality frame selection.

Otoscopy videos frequently contain frames affected by motion blur, incomplete visualization of the tympanic membrane, or occlusion by cerumen or debris. To ensure that model training and evaluation were based on diagnostically informative images, an automated quality-control procedure was applied to select high-quality frames from each video.

Frame quality was quantified using three metrics derived from the LUCID framework [19]. For all three metrics, higher values indicate better image quality:

1. **Blur Score**, which quantifies image sharpness and penalizes motion or focus artifacts (higher values correspond to sharper images).
2. **Eardrum View Score**, which measures the visibility and clarity of the tympanic membrane within the frame (higher values indicate clearer visualization).
3. **Coverage Score**, which evaluates the proportion of the frame occupied by the tympanic membrane region (higher values indicate greater anatomical coverage).

For each video, these three features were computed for all frames. The resulting feature vectors were clustered using K-means clustering with K = 3. We selected K = 3 a priori to reflect clinically intuitive low-, intermediate-, and high-quality frame groups; exploratory analyses showed that this choice produced stable and interpretable clusters across videos. The high-quality cluster was characterized by substantially higher coverage and eardrum visibility compared with the other clusters, while maintaining comparably high image sharpness. The numerical cluster centers are summarized in Table 3.

**Table 3:** Cluster centers used for automated frame-quality classification. Higher values indicate better image quality for all three metrics. Frames assigned to the high-quality cluster were retained for model training and evaluation.

Cluster	Coverage Score	Eardrum View Score	Blur Score
High	0.836	0.946	8.006
Intermediate	0.734	0.408	8.138
Low	0.408	0.550	0.069

Only frames assigned to the high-quality cluster were retained for subsequent model training and evaluation. The selected frames constitute the datasets summarized in Tables 1 and 2. For each video, all frames assigned to the high-quality cluster were retained, allowing video-level diagnosis to be based on multiple diagnostically informative frames rather than a single selected image. For validation and external test cohorts, high-quality frame selection was used only to select diagnostically informative frames for model input and did not constitute additional video-level exclusion. Thus, OSU-validation videos and VUMC paired test videos remained in the analysis cohort after frame selection.

#### Data augmentation.

To improve robustness to cross-center domain variability, on-the-fly data augmentation was applied only to training frames; validation and test images were not augmented. All frames were resized to 224 × 224 pixels. Mild geometric and photometric perturbations were applied to simulate acquisition variability, including random affine transformation (rotation within ±8°, translation up to 5%, shear up to 5°), horizontal flipping (probability 0.5), and color jitter (brightness ±0.25, saturation ±0.15).

To mimic real-world otoscopy acquisition artifacts, we introduced domain-specific augmentations. Border-connected low-luminance regions were optionally replaced with canal-like flesh tones using a feathered mask, preserving dark structures within the tympanic membrane. Random zoom-out augmentation was applied by shrinking the image content and placing it on a dark background to simulate variations in the field of view and working distance. Additional augmentations included soft elliptical canal occlusion and simulated specular reflection to improve robustness to partial visualization and illumination artifacts.

To encourage the model to focus on diagnostically relevant anatomy, a center-preserving circular mask was applied during training as an attention regularization strategy. This operation suppresses peripheral background information and promotes reliance on tympanic membrane features. This augmentation was not applied during validation or testing.

#### Frame-level classification and video-level aggregation.

A convolutional neural network based on ResNet-50 was used for frame-level classification. The model was trained using all high-quality frames from the training dataset. For each input frame, the network outputs a vector of logits (unnormalized scores) for all diagnostic categories. These logits are converted into class probabilities using the softmax function.

Video-level predictions were obtained by averaging the frame-level class probabilities across all selected frames within each video. Specifically, for a video with N frames, the predicted probability for class c is computed as:


$$\begin{array}{c} P_{c}^{video}=\frac{\text{1}}{N}\sum_{i=\text{1}}^{N}P_{c}^{\left(i\right)}, \end{array}$$ (1)

Where ${\text{P}}_{\text{c}}^{\left(i\right)}$ denotes the softmax probability of class c for the i-th frame. This aggregation strategy does not require every selected frame to show the abnormality. Rather, it assumes that the retained high-quality frames collectively contain sufficient diagnostic evidence, and averaging frame-level probabilities provides a stable video-level prediction while reducing the influence of occasional ambiguous frames.

During inference, predictions were generated at the video level to reflect real-world clinical interpretation. The final predicted class for each video was determined as the class with the highest averaged probability.

#### Confidence-guided rule-based decision fusion with tympanometry.

To improve diagnostic robustness in visually ambiguous cases, we incorporated conventional 226 Hz tympanometry as a physiologically grounded secondary modality using decision-level fusion. This module was not designed as a jointly trained multimodal neural network; instead, it applies clinically interpretable tympanometry rules to refine low-confidence CNN predictions. Multimodal decision fusion was applied exclusively to the VUMC-external test dataset, where paired tympanometry measurements were available from the same clinical visit and corresponding ear examination.

**Tympanometry rule mapping.** Tympanometry measurements were interpreted using established clinical criteria based on tympanogram curve type (“Curve Type”) and ear canal volume (ECV). Curve types were normalized to uppercase values, and ECV values were extracted from free-text entries using numeric parsing.

The following mapping was applied:

- Type A (including A/As/Ad) → Normal middle ear function
- Type C → Retraction physiology (negative middle ear pressure)
- Type B with ECV ≤ 2.0 mL → Effusion
- Type B with ECV > 2.0 mL → Perforation
- OPEN → Perforation

This rule-based mapping was intended as a conservative decision-level adjunct to video-based diagnosis rather than a complete clinical tympanometry interpretation system. For Type B/flat tympanograms, ECV was used to distinguish non-large ECV patterns, interpreted as effusion physiology, from large ECV or open patterns, interpreted as perforation or an open ventilation pathway. The ECV cutoff of >2.0 mL was selected as a simple, clinically interpretable large-volume threshold and was not optimized on test labels. Device-specific ECV calibration metadata were not uniformly available in the retrospective records; therefore, device-specific calibration was not attempted.

The VUMC external paired cohort included adult ear-level examinations only (median age, 51.0 years; IQR, 33.0–65.0; range, 18–85). By age group, 43 examinations were from patients aged 18–44 years, 33 from patients aged 45–64 years, and 28 from patients aged >=65 years. No pediatric cases were included; therefore, this cohort does not support evaluation of pediatric or age-specific ECV thresholds.

All 104 examinations had a recorded 226 Hz tympanogram curve type: 43 Type A, 4 Type As, 4 Type Ad, 17 Type C, 21 Type B/flat, and 15 Open tympanograms. Among the 21 Type B/flat tympanograms, 15 had ECV <=2.0 mL, 5 had ECV > 2.0 mL, and 1 had missing ECV. Six examinations had acquisition-related notes suggesting potential interpretability limitations, including failed seal or equipment/computer issues. Tympanograms with missing ECV required for Type B subtyping or acquisition-related interpretability concerns were handled conservatively and were not used for additional fine-grained tympanometric subtyping beyond the predefined rule-mapping strategy.

**Confidence-triggered rule-guided refinement.** The CNN model first generated a video-level prediction from otoscopy frames using averaged frame-level probabilities. Decision-level fusion was then applied using a confidence-triggered strategy that mirrors clinical decision-making.

Specifically, refinement was performed only when:

- The CNN did not predict tympanostomy tube or tympanosclerosis, and
- The maximum predicted class probability was below an operating threshold τ.

Because paired tympanometry was available only in the VUMC external paired cohort, an independent multimodal validation set was not available for prespecifying or optimizing τ. Therefore, the confidence-threshold analysis was treated as exploratory. We evaluated a range of thresholds from 0.50 to 1.00 to characterize the sensitivity of the hybrid decision strategy to τ. The threshold of 0.90 was reported as the best observed operating point in this exploratory analysis, rather than as a threshold validated on an independent multimodal validation set. Accordingly, performance at τ = 0.90 should be interpreted as hypothesis-generating and requiring confirmation in future paired multimodal validation cohorts with prespecified thresholds.

The overall design of applying tympanometry only to visually uncertain cases was motivated by clinical considerations, whereas the specific numerical threshold was selected from an exploratory threshold analysis in the external paired cohort. Tympanostomy tube and tympanosclerosis predictions were not refined using tympanometry because these categories are primarily defined by visible structural findings on otoscopy. In this study, tympanometry was used as an adjunct to refine visually uncertain predictions, not as a replacement for visually distinctive otoscopic findings. Tympanometry can be difficult to interpret for these categories because patent and blocked tympanostomy tubes can produce different tympanometric patterns, tympanosclerosis may alter tympanic membrane stiffness without defining a unique tympanometric class, and mixed pathology can further complicate interpretation. Therefore, when the CNN predicted tympanostomy tube or tympanosclerosis, the video-based prediction was retained to avoid replacing visually distinctive findings with potentially nonspecific tympanometric patterns. Algorithm 1 shows this process. We refer to this approach as the confidence-guided hybrid decision-fusion model, or simply the hybrid model.

Algorithm 1. Confidence-guided hybrid multimodal diagnosis

Input:

  V: otoscopy video

  T: tympanometry measurement

  τ: confidence threshold

Output:

  y_final: final diagnostic label

1. Compute CNN-based video prediction from V:

   $p_{video}$ = $\frac{\text{1}}{N}\sum_{i=\text{1}}^{N}Softmax\left(CNN\left(x_{i}\right)\;\right)$

   $y_{cnn}$ = $argmax_{c}(p_{video})$

   $s=\underset{c}{\text{max}}p_{video,c}$

2. If s ≥ τ or y_cnn = “tube” or y_cnn = “tympanosclerosis” then

  y_final = y_cnn

 Else

  y_ final = TympanometryDecision(T)

 End if

3. Return y_final

#### Model training details.

The frame-level classification model was trained using supervised learning with categorical cross-entropy loss. Optimization was performed using the Adam optimizer with an initial learning rate of $\text{1}.\text{0}\times {\text{10}}^{-\text{5}}$.

Training was conducted for a maximum of 150 epochs. To prevent overfitting and improve model generalization, early stopping was applied based on validation performance. Specifically, training was terminated if the validation overall accuracy did not improve for 20 consecutive epochs (patience = 20), with no minimum improvement threshold (min_delta = 0.0). The best-performing model checkpoint was selected based on maximum validation overall accuracy.

All experiments were implemented using the PyTorch Lightning framework and trained on NVIDIA A100 GPUs with 80 GB of memory.

#### Statistical analysis.

Model performance was evaluated at the video level using the clinician-assigned reference standard described above. Each ear examination/video was assigned a single consensus primary diagnosis, focusing on the most clinically relevant or dominant abnormality. In cases with multiple coexisting findings, only the primary diagnosis was considered for evaluation, while secondary abnormalities were not included as additional ground-truth labels. For example, ears treated with tympanostomy tubes may also present with tympanosclerosis; in such cases, the sample was categorized as “tympanostomy tube” only, and tympanosclerosis was not considered a separate ground-truth label for the single-label evaluation.

In the primary-label evaluation, a prediction was considered correct if the predicted class matched the clinician-assigned primary diagnosis. Per-class performance was evaluated using a one-versus-rest approach. For each diagnostic category, accuracy, sensitivity, and specificity were calculated. Balanced accuracy was computed as the average of sensitivity and specificity. All statistical analyses were performed in Python using the *statsmodels* library.

Because paired tympanometry was available only in the VUMC external paired cohort, an independent paired multimodal validation set was not available for prespecifying or optimizing τ. Therefore, the confidence-threshold analysis was treated as exploratory. We evaluated thresholds from 0.50 to 1.00 to characterize the behavior of the hybrid decision-fusion strategy across operating points. The threshold of τ = 0.90 was reported as the best observed operating point in this exploratory analysis, rather than as a threshold validated on an independent multimodal validation set. Accordingly, performance at τ = 0.90 should be interpreted as hypothesis-generating and may be optimistic due to threshold selection within the external paired cohort. We did not estimate category-specific or institution-specific thresholds because the paired cohort was not large enough to support reliable subgroup-level calibration. Future studies with larger paired multimodal validation cohorts should prespecify or calibrate operating thresholds before final external testing.

To evaluate systematic disagreement between expert labels and model predictions, Bowker’s test of symmetry and the Stuart-Maxwell test of marginal homogeneity were applied to the multi-class confusion matrices for the CNN-only and hybrid models. These tests were used to determine whether model errors demonstrated directional misclassification patterns relative to expert diagnosis. For each diagnostic category, one-versus-rest AUROC values were calculated for the CNN-only and hybrid models, and paired AUROC curves were compared using DeLong’s test. Bonferroni correction was applied to account for multiple comparisons across diagnostic categories.

To quantify statistical uncertainty, two-sided 95% confidence intervals (CIs) were reported for all primary performance estimates. Overall accuracy and one-versus-rest per-class accuracy, sensitivity, and specificity were calculated with Wilson binomial confidence intervals. Balanced accuracy was defined as the average of sensitivity and specificity, and its 95% CI was estimated using nonparametric bootstrap resampling of the external test set. The absolute improvement in overall accuracy between the hybrid model and the CNN-only model was evaluated using paired bootstrap resampling, preserving the paired nature of predictions generated on the same test examinations. For AUROC analyses, one-versus-rest AUROC estimates and paired AUROC differences were reported with 95% confidence intervals where applicable; DeLong tests were interpreted as exploratory because several diagnostic categories had small positive sample sizes.

## Results

### Overall diagnostic performance

Because the VUMC external paired cohort did not include AOM cases, all external paired-cohort performance analyses were restricted to the six diagnostic categories represented in that cohort: effusion, normal, perforation, retraction, tympanostomy tube presence, and tympanosclerosis. The overall diagnostic performance of the proposed system is summarized in Table 4. In the external paired test cohort, the CNN-only otoscopy video model achieved an overall accuracy of 63.46% (95% CI, 53.88–72.08), while tympanometry alone achieved an accuracy of 54.81% (95% CI, 45.24–64.03).

**Table 4:** Overall diagnostic performance in test set of CNN-only, tympanometry-only, and hybrid models.

Metric	CNN Only	Tymp Only	Hybrid	Hybrid vs CNN, absolute difference (percentage points).
Ear-level overall accuracy	63.46% (95% CI, 53.88–72.08)	54.81% (95% CI, 45.24–64.03)	81.73% (95% CI, 73.22–87.98)	+18.27 (95% CI, 9.62–26.92)

Note. Values are percentages with 95% confidence intervals. Accuracy CIs were calculated using Wilson binomial intervals. The CI for Hybrid vs CNN represents a paired bootstrap CI for the absolute accuracy difference.

In the exploratory external paired-cohort analysis, the hybrid decision-fusion framework, which integrates CNN predictions with tympanometry-based rule refinement for low-confidence cases, was associated with improved diagnostic performance. At the best observed exploratory threshold of 0.90, the hybrid model achieved an overall accuracy of 81.73% (95% CI, 73.22–87.98), corresponding to an absolute improvement of 18.27 percentage points over the CNN-only model (95% paired bootstrap CI, 9.62–26.92). Because the operating threshold was selected from exploratory analysis in the same external paired cohort, this result should be interpreted as an observed improvement supporting the feasibility of the confidence-triggered fusion strategy, rather than definitive validation of a fixed threshold. The magnitude of improvement should be confirmed in larger prospective paired cohorts with prespecified thresholds.

These results suggest that combining visual otoscopy analysis with physiological tympanometry may improve diagnostic performance in this external paired cohort. Because the paired multimodal test cohort was modest in size and the operating threshold was exploratory, the magnitude of improvement should be confirmed in larger prospective paired cohorts.

### Per-class diagnostic performance

Per-class performance of the final hybrid model is summarized in Table 5, and complete per-class results for the CNN-only, tympanometry-only, and hybrid models are provided in Table B in S1 Text. Metrics were calculated using one-versus-rest evaluation and are presented with 95% confidence intervals. Overall, the hybrid framework showed improved sensitivity for several clinically important pathologies while maintaining high specificity, although confidence intervals were wide for categories with small numbers of positive cases.

**Table 5:** Per-class performance of the hybrid model on the external paired test cohort.

Class	n	Accuracy	Sensitivity	Specificity	Balanced accuracy
Effusion	11	91.35 (84.37–95.38)	72.73 (43.44–90.25)	93.55 (86.63–97.01)	83.14 (67.98–96.91)
Normal	35	80.77 (72.15–87.19)	94.29 (81.39–98.42)	73.91 (62.49–82.81)	84.10 (77.35–90.23)
Perforation	20	94.23 (87.98–97.33)	90.00 (69.90–97.21)	95.24 (88.39–98.13)	92.62 (84.78–98.80)
Retraction	20	83.65 (75.37–89.54)	60.00 (38.66–78.12)	89.29 (80.88–94.26)	74.64 (62.86–85.69)
Tympanostomy tube	10	97.12 (91.86–99.01)	80.00 (49.02–94.33)	98.94 (94.22–99.81)	89.47 (74.49–100.00)
Tympanosclerosis	8	96.15 (90.53–98.49)	75.00 (40.93–92.85)	97.92 (92.72–99.43)	86.46 (68.75–99.50)

Note. Values are percentages with 95% confidence intervals. Per-class metrics were calculated using one-versus-rest evaluation. Accuracy, sensitivity, and specificity CIs were calculated using Wilson binomial intervals. Balanced accuracy CIs were estimated using bootstrap resampling.

Because several diagnostic categories contained few positive cases, sensitivity estimates should be interpreted together with their numerators and denominators. For example, the hybrid sensitivity for tympanosclerosis was 75.00% but corresponded to 6 of 8 positive cases, compared with 4 of 8 for the CNN-only model. Similarly, the hybrid sensitivity for tympanostomy tube was 80.00%, corresponding to 8 of 10 positive cases. Therefore, these per-class changes should be interpreted as descriptive and hypothesis-generating rather than statistically stable estimates.

For middle ear effusion, hybrid sensitivity was 72.73% (95% CI, 43.44–90.25), compared with 36.36% (95% CI, 15.17–64.62) for the CNN-only model and 54.55% (95% CI, 28.01–78.73) for tympanometry alone. Hybrid specificity for effusion remained high at 93.55% (95% CI, 86.63–97.01).

For tympanic membrane perforation, the hybrid model achieved 90.00% sensitivity (95% CI, 69.90–97.21) and 95.24% specificity (95% CI, 88.39–98.13), with a balanced accuracy of 92.62% (95% CI, 84.78–98.80). For retraction, CNN-only prediction did not detect positive cases, with a sensitivity of 0.00% (95% CI, 0.00–16.11), whereas the hybrid model achieved a sensitivity of 60.00% (95% CI, 38.66–78.12) and specificity of 89.29% (95% CI, 80.88–94.26).

For tympanostomy tubes and tympanosclerosis, the hybrid model achieved sensitivity estimates of 80.00% (95% CI, 49.02–94.33) and 75.00% (95% CI, 40.93–92.85), respectively, while maintaining high specificity. For normal ears, the hybrid model achieved high sensitivity, 94.29% (95% CI, 81.39–98.42), but lower specificity, 73.91% (95% CI, 62.49–82.81), reflecting a tradeoff between preserving normal-ear specificity and improving sensitivity for pathological findings.

### Patient-level sensitivity analysis

The VUMC external paired cohort included 104 ear-level examinations from 76 unique patients. Among these, 28 patients contributed bilateral ear examinations, corresponding to 56 ear-level examinations, and 48 patients contributed one ear examination. There were 56 right-ear and 48 left-ear examinations.

In the patient-clustered bootstrap analysis, the hybrid model achieved an overall accuracy of 81.73% (95% CI: 74.87–88.49), compared with 63.46% (95% CI: 56.46–71.97) for the CNN-only model and 54.81% (95% CI: 43.05–62.52) for tympanometry alone. The paired accuracy improvement of the hybrid model relative to the CNN-only model was 18.27 percentage points (95% CI: 9.61–27.59).

In the one-ear-per-patient sensitivity analysis using 100 repeated random selections of one ear per patient, the hybrid model remained higher than the CNN-only model. The mean overall accuracy was 82.64% for the hybrid model, 65.05% for the CNN-only model, and 56.05% for tympanometry alone. The mean paired improvement of the hybrid model over the CNN-only model was 17.59 percentage points. These results indicate that the observed improvement was not solely driven by inclusion of bilateral ear examinations.

### Confusion matrix analysis

The hybrid-model confusion matrix is shown in Table 6. Normal ears were frequently classified correctly, with 33 of 35 normal cases predicted as normal. Structural abnormalities also showed relatively high observed detection in this cohort, including 18 of 20 perforation cases and 6 of 8 tympanosclerosis cases. Residual errors were concentrated in clinically overlapping categories. Effusion errors were mainly classified as normal (3 of 11), whereas retraction errors were mainly classified as normal (5 of 20) or perforation (2 of 20). These patterns are clinically plausible because mild effusion, retraction, and tympanic membrane structural changes can have subtle or overlapping otoscopic appearances under single-label evaluation. Given the modest cohort size, these error patterns should be interpreted descriptively rather than as definitive evidence of class-specific behavior.

**Table 6:** Confusion matrix of the hybrid multimodal model on the external test dataset.

Ground Truth	normal	effusion	perforation	retraction	tube	tympanosclerosis
normal	33	0	1	1	0	0
effusion	3	8	0	0	0	0
perforation	0	0	18	1	0	1
retraction	5	0	2	12	0	1
tube	1	1	0	0	8	0
tympanosclerosis	0	1	0	1	0	6

### Statistical comparison between CNN-only and hybrid models

Additional statistical analyses were conducted to evaluate whether multimodal integration reduced systematic diagnostic disagreement and changed disease-level discrimination. Bowker’s test of symmetry demonstrated significant asymmetry between expert labels and CNN-only predictions (p = 0.0052), and the Stuart-Maxwell test showed significant marginal inhomogeneity ($\text{p}=\text{1}.\text{53 x }{\text{10}}^{-\text{5}}$). In contrast, no significant asymmetry or marginal inhomogeneity was observed for the hybrid model (Bowker p = 0.753; Stuart-Maxwell p = 0.309), suggesting fewer directional disagreement patterns in this cohort.

**Fig 3:**
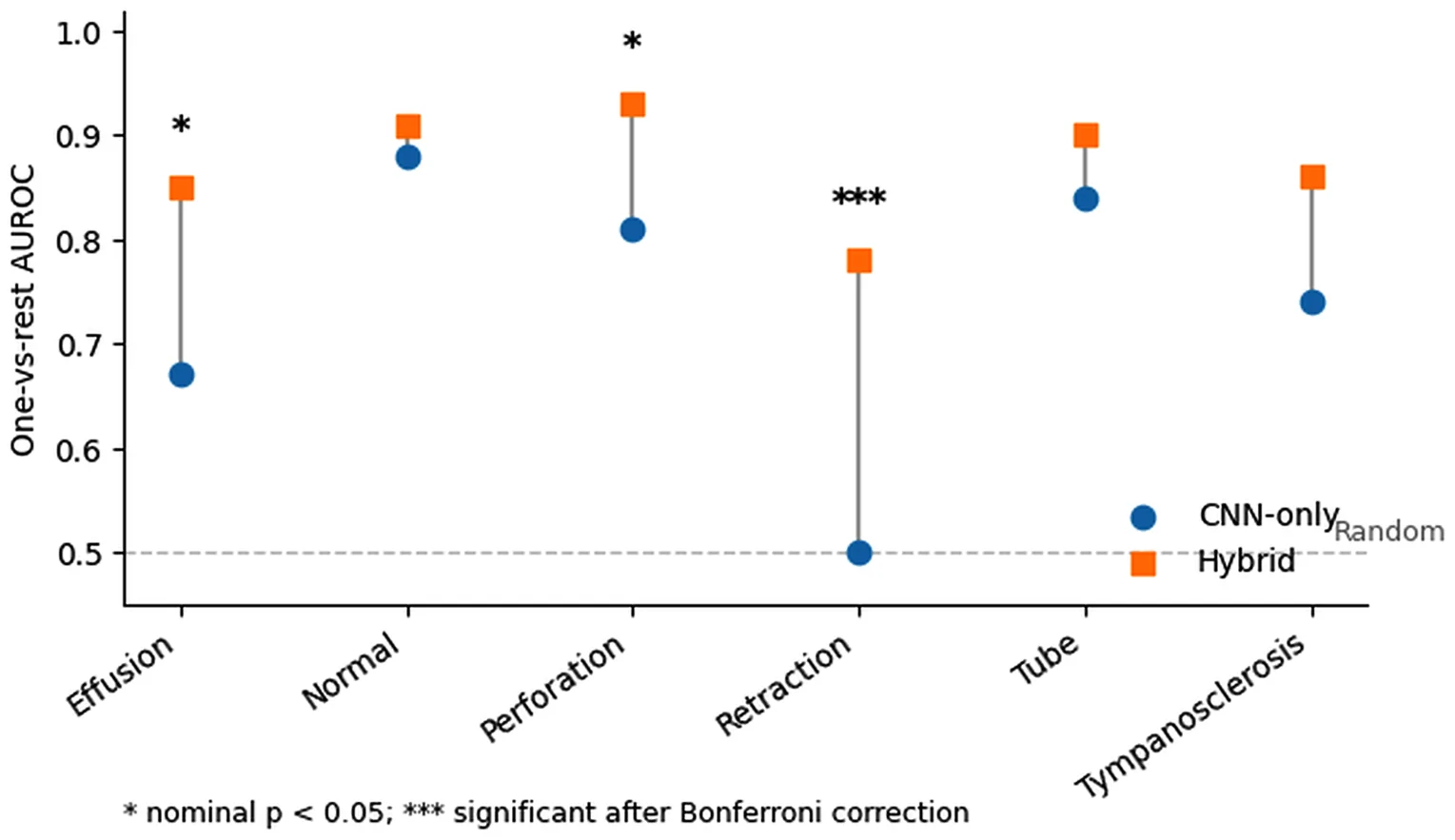
One-versus-rest AUROC comparison between CNN-only and hybrid models.

One-versus-rest AUROC values were numerically higher for the hybrid model across all diagnostic categories. Paired AUROC comparisons were evaluated using DeLong’s test, and 95% CIs for paired AUROC differences (Hybrid minus CNN-only) are reported in Table I in S1 Text. The largest observed AUROC increase was for retraction (0.50 to 0.78; DeLong p=$\text{7}.\text{8}\text{ x }{\text{10}}^{-\text{7}}$), which remained significant after Bonferroni correction. Effusion and perforation showed nominally significant AUROC increases, but these did not meet the Bonferroni-adjusted threshold. Because the external paired cohort was modest and some categories had small positive sample sizes, these AUROC comparisons should be interpreted as exploratory (Fig 3).

### Exploratory analysis: Confidence threshold

We performed an exploratory analysis to evaluate the effect of the confidence threshold used to trigger tympanometry-based refinement. Because paired tympanometry was available only in the VUMC external paired cohort, no independent paired multimodal validation set was available to prespecify the threshold. Therefore, this analysis was used to characterize the behavior of the hybrid decision-fusion strategy across operating points rather than to establish a definitively validated threshold (Table 7).

**Table 7:** Statistical comparison between CNN-only and hybrid models.

Analysis	CNN-only	Hybrid	Interpretation
Bowker’s test of symmetry	p = 0.0052	p = 0.753	CNN showed directional misclassification; Hybrid did not
Stuart-Maxwell test	p=1.53×10^-5^	p = 0.309	CNN showed marginal disagreement; Hybrid did not
Effusion AUROC	0.67	0.85, p = 0.015	Improved discrimination
Normal AUROC	0.88	0.91, p = 0.302	Similar performance
Perforation AUROC	0.81	0.93, p = 0.049	Improved discrimination
Retraction AUROC	0.50	0.78, p = 7.8 × 10^-7^	Significant after Bonferroni correction
Tube AUROC	0.84	0.90, p = 0.271	Similar performance
Tympanosclerosis AUROC	0.74	0.86, p = 0.144	Nonsignificant improvement

Because multiple thresholds were evaluated in the same external paired cohort and τ = 0.90 was the best observed operating point, the reported 81.73% accuracy may be optimistic and should be interpreted as hypothesis-generating. Importantly, the observed improvement was not limited to τ = 0.90. Across thresholds from 0.60 to 0.95, the hybrid model consistently outperformed the CNN-only baseline by more than 10 percentage points in overall accuracy. This pattern suggests that, within this cohort, the benefit of confidence-triggered tympanometry refinement reflected the overall decision-fusion structure rather than a single isolated threshold value.

These findings support the feasibility of the confidence-triggered decision-fusion strategy in this external paired cohort. However, the optimal numerical threshold remains exploratory and should be prespecified, recalibrated, and validated in future independent paired multimodal cohorts (Table 8).

**Table 8:** Exploratory analysis of the confidence threshold used for multimodal gating in the external paired cohort.

Confidence Threshold	Hybrid Accuracy	Hybrid vs CNN, absolute difference (percentage points).
0.50	73.08%	+9.62
0.60	75.00%	+11.54
0.70	76.92%	+13.46
0.75	77.88%	+14.42
0.80	78.85%	+15.38
0.85	80.77%	+17.31
**0.90**	**81.73%**	**+18.27**
0.95	78.35%	+14.88
1.00	54.81%	−8.65

**Note.** This threshold analysis was exploratory because no independent paired multimodal validation set was available. The threshold of τ = 0.90 represents the best observed operating point in the external paired cohort and should not be interpreted as a prespecified or independently validated threshold.

### Ablation study: Effect of data processing strategies

To assess the impact of data preprocessing and training strategies, we conducted additional ablation experiments evaluating the effects of data augmentation and high-quality frame selection. The results are summarized in Table 9.

**Table 9:** Effect of data augmentation and frame selection on overall diagnostic performance.

Model Setting	CNN Only	Tympanometry Only	Hybrid	absolute difference (percentage points)
**Without augmentation**	56.73%	54.81%	74.04%	+17.31%
**Without frame selection**	55.72%	54.81%	70.19%	+14.47%
**Proposed method**	**63.46%**	54.81%	**81.73%**	**+18.27%**

Without augmentation, the CNN-only model achieved 56.73% accuracy, and the hybrid model achieved 74.04% accuracy, representing a 17.31% point improvement over CNN alone.

When frame selection was not applied, the hybrid system achieved 70.19% accuracy, indicating that removing low-quality frames contributes to improved diagnostic performance.

The proposed method, which combines both augmentation and high-quality frame selection, achieved the highest observed overall accuracy in this external test cohort, suggesting that both preprocessing strategies contributed to improved model performance.

## Discussion

This study presents OtoTymp-AI, a confidence-guided multimodal AI framework for diagnosing middle ear conditions by integrating otoscopy video analysis with conventional 226 Hz tympanometry. The framework was evaluated within a multi-center otoscopy study, with model development and validation performed using videos from independent institutions and multimodal evaluation performed on the VUMC-external test dataset, where paired tympanometry was available. This distinction is important because the otoscopy component was developed, selected, and externally tested using institutionally separated cohorts, whereas the video-plus-tympanometry component was evaluated in a single independent external paired cohort from VUMC.

The results suggest that combining anatomical video information with physiological tympanometry may improve diagnostic performance compared with either modality alone in this external paired cohort. The CNN-only model achieved moderate performance, whereas the hybrid model achieved a higher observed accuracy in the exploratory threshold analysis. At the best observed threshold of 0.90, accuracy increased from 63.46% to 81.73%; however, because this threshold was selected from the exploratory analysis rather than prespecified in an independent multimodal validation cohort, this result should be interpreted as hypothesis-generating.

These findings are consistent with clinical diagnostic reasoning. Otoscopy provides anatomical information, including tympanic membrane color, opacity, position, and structural changes, while tympanometry provides functional information about middle ear pressure, compliance, and ear canal volume. The confidence-guided design allows the model to preserve high-confidence visual predictions while using tympanometry to refine uncertain cases. This approach also improves interpretability because each refinement step follows clinically meaningful tympanometry rules rather than relying on an opaque end-to-end fusion model.

The statistical analyses provided additional exploratory evidence supporting the potential value of multimodal integration, while also highlighting the need for cautious interpretation in small per-class subgroups. The CNN-only model showed significant directional disagreement with expert labels, while the hybrid model did not show significant asymmetry or marginal disagreement. In addition, one-versus-rest AUROC improved across all diagnostic categories, with the strongest improvement observed for retraction. These results suggest that tympanometry not only increases the number of correct predictions but also helps reduce systematic error patterns in visually challenging disease categories.

The modest size of the paired multimodal test cohort should be interpreted in the context of the difficulty of collecting ear-specific otoscopy videos with paired tympanometry from the same clinical encounter. Many AI otoscopy studies rely on visual data alone, whereas paired tympanometry requires additional clinical testing, encounter-level matching, and ear-specific modality alignment. To our knowledge, relatively few studies have evaluated AI-based otoscopy video diagnosis together with independently paired tympanometry in an external clinical cohort. Thus, despite the limited paired cohort size and absence of AOM cases, this study provides an initial feasibility evaluation of how anatomical video information and physiological tympanometry may complement each other for selected middle-ear conditions represented in this cohort.

This study has several limitations. First, the external paired test cohort was modest in size and did not include AOM cases, although AOM was present in the training and validation datasets. This limits the clinical completeness of the external paired-cohort evaluation and prevents conclusions about AOM performance in the independent paired cohort. Several diagnostic categories also had small positive sample sizes, including effusion, tympanostomy tube, and tympanosclerosis, resulting in wide confidence intervals. Therefore, per-class findings should be interpreted as exploratory and hypothesis-generating until validated in larger paired multimodal cohorts.

Second, paired tympanometry was available only in the VUMC external cohort. Accordingly, the video-plus-tympanometry analysis should be interpreted as an external paired-cohort feasibility evaluation rather than multi-center validation of the full multimodal framework. Future studies should validate the decision-fusion strategy using larger paired otoscopy-tympanometry datasets from multiple institutions.

Third, the current multimodal fusion strategy is a rule-based, decision-level approach rather than a jointly trained multimodal representation-learning model. Its performance depends on the appropriateness of the tympanometry mapping and confidence gate. The threshold of 0.90 was selected through exploratory analysis in the external paired cohort, because no independent paired multimodal validation set was available. Therefore, this operating point should not be considered prespecified or independently validated, and the corresponding accuracy estimate may be optimistic. Future studies should prespecify thresholds using independent paired multimodal validation cohorts and evaluate category-specific or institution-specific recalibration.

Fourth, the tympanometry mapping was intentionally simplified. It does not fully address patent versus blocked tympanostomy tubes, pediatric or age-specific ECV thresholds, mixed pathology, tympanosclerosis-related stiffness changes, or device-specific calibration differences. The VUMC paired cohort contained adult examinations only, so pediatric thresholds could not be evaluated. Future work should test calibrated, age-aware, and probabilistic tympanometry integration strategies in larger paired cohorts.

Fifth, cohort curation and quality filtering may introduce selection or spectrum bias. Although the OSU validation cohort and VUMC paired cohort were not subjected to additional case-level exclusion after cohort assignment, training-cohort diagnostic agreement screening and video-level quality filtering may underrepresent diagnostically ambiguous or severely low-quality otoscopy videos. This may limit generalizability to fully unfiltered primary care settings, where poor visualization, obstruction, motion artifact, and clinical ambiguity are common.

Sixth, some patients contributed bilateral ear examinations, which may introduce within-patient correlation. Patient-clustered bootstrap and one-ear-per-patient sensitivity analyses showed that the observed hybrid-vs-CNN difference was not solely driven by bilateral-ear inclusion. However, these analyses do not prove independence between ears, and future larger studies should consider patient-level or clustered modeling approaches prospectively.

Seventh, although tympanometry was not used to assign the clinician reference labels, the reference standard was still based on expert interpretation of otoscopic video findings rather than a fully independent composite adjudication standard. Video-based expert labels may be imperfect in subtle, overlapping, or mixed middle-ear findings. Future prospective studies should use prespecified adjudication procedures and, where appropriate, independent clinical follow-up or composite diagnostic standards.

Finally, this study did not compare model performance with primary care clinicians, trainees, otolaryngologists, or standard clinical workflows. Therefore, claims about clinical impact, workflow improvement, or deployment readiness remain speculative. Prospective studies with clinician-comparator arms are needed to evaluate diagnostic accuracy, confidence, workflow effects, and patient-level outcomes before clinical implementation.

Future work should evaluate prospective clinical deployment, larger multi-center multimodal cohorts, and adaptive fusion strategies that preserve interpretability while improving generalizability.

## Conclusion

In this study, we proposed OtoTymp-AI, a clinically interpretable confidence-guided decision-fusion framework that integrates deep learning-based otoscopy video analysis with conventional tympanometry. In one external paired VUMC cohort, the hybrid approach showed higher observed accuracy than the video-only CNN model, with uncertainty quantified using 95% confidence intervals. Because the operating threshold was exploratory, the paired cohort was modest in size, and no clinician-comparator or prospective workflow evaluation was performed, these findings should be interpreted as technical feasibility evidence rather than definitive clinical validation. Larger prospective paired multimodal cohorts with sufficient AOM representation, prespecified thresholds, and clinician-comparator evaluation are needed to confirm diagnostic performance and generalizability.

## Supporting information

S1 Text Supplementary methods and results. This file includes supplementary details on cohort curation, patient-level sensitivity analyses, systematic diagnostic disagreement testing, one-versus-rest AUROC analysis, complete supplementary tables supporting the main results, and supplementary one-versus-rest ROC curves.
(DOCX)
