# Supplementary material for: Confidence-guided integration of otoscopy videos and tympanometry for middle ear disease diagnosis: A multi-center otoscopy study with external paired-cohort evaluation
Source: PLoS One. 2026 Sep 1;21(9):e0357492. doi: 10.1371/journal.pone.0357492 (PMC13533359; doi:10.1371/journal.pone.0357492)
Supplement: Supplementary file 1 [file pone.0357492.s001.docx]

S1 Text. Supplementary methods and results

## Supplementary Methods

### Cohort curation and exclusion reporting

Institution-level cohort curation and retention were summarized to clarify how many cases were excluded during dataset construction. The NCH-training dataset underwent diagnostic agreement screening and clinician-defined video-level quality filtering before automated high-quality frame selection. The OSU-validation dataset and the VUMC external paired cohort were not subjected to additional case-level exclusion after cohort assignment. Category-level exclusion counts were not available for the validation and external paired test cohorts because no additional case-level exclusions were applied to those cohorts after cohort assignment.

### Patient-level sensitivity analysis for bilateral-ear contribution

Because some patients contributed bilateral ear examinations, patient-level sensitivity analyses were performed rather than relying on ear-level independence assumptions alone. Patient identifiers and ear side were extracted from the examination ID, where the final character denoted right or left ear. We first summarized the number of ear-level examinations, unique patients, unilateral-ear contributors, bilateral-ear contributors, and right- versus left-ear examinations.

We then performed patient-clustered bootstrap resampling. In each bootstrap replicate, patients were sampled with replacement, and all available ear-level examinations from the sampled patients were included. This approach preserves the clustering structure of bilateral ear examinations within patients. Overall accuracy and paired accuracy improvement between the hybrid and CNN-only models were recalculated in each bootstrap replicate, and 95% confidence intervals were estimated from the 2.5th and 97.5th percentiles.

As an additional sensitivity analysis, we repeatedly selected one ear examination at random from each patient and recalculated overall accuracy for the CNN-only, tympanometry-only, and hybrid models, as well as the paired accuracy improvement of the hybrid model relative to the CNN-only model. The resulting percentile range reflects sensitivity to random ear selection and was not interpreted as proof of statistical independence between ears.

### Assessment of systematic diagnostic disagreement

To evaluate whether model predictions demonstrated systematic directional disagreement relative to expert diagnosis, Bowker’s test of symmetry and the Stuart-Maxwell test of marginal homogeneity were applied to the multiclass contingency tables comparing expert labels with model predictions.

Bowker’s test evaluates whether off-diagonal misclassification patterns are symmetric in a square contingency table. In this study, a significant Bowker test indicates that model errors were directionally imbalanced; for example, one diagnostic class may have been preferentially misclassified as another class more often than the reverse pattern. The Stuart-Maxwell test evaluates marginal homogeneity between expert labels and model predictions. A significant Stuart-Maxwell test indicates that the overall distribution of predicted labels differs from the distribution of expert labels.

These tests were applied separately to the CNN-only and hybrid models. Non-significant results for the hybrid model were interpreted as evidence that multimodal integration reduced systematic directional misclassification relative to the CNN-only model.

### One-versus-rest AUROC analysis and DeLong test

Disease-level discriminative performance was evaluated using one-versus-rest area under the receiver operating characteristic curve values. For each diagnostic category, cases belonging to that category were treated as positive, and all other diagnostic categories were treated as negative.

AUROC values were calculated separately for the CNN-only and hybrid models. Paired AUROC curves were compared using DeLong’s test. Because six diagnostic categories were evaluated, Bonferroni correction was applied to account for multiple comparisons, resulting in an adjusted significance threshold of p < 0.0083.

Statistical comparison analyses were performed in the subset of cases with complete expert labels and model outputs available for both CNN-only and hybrid model comparisons. This complete-case subset included 104 ear-level examinations.

## Supplementary Results

### Cohort curation and retention

Case-level cohort curation and retention by institution are summarized in Table A in S1 Text. The curation summary distinguishes training-cohort exclusions from validation and external paired test cohorts, where no additional case-level exclusions were applied after cohort assignment.

Table A in S1 Text. Case-level cohort curation and retention by institution.

| **Institution / split** | **Initial videos** | **Diagnostic disagreement excluded** | **Poor visualization excluded** | **Additional case-level exclusion after paired tympanometry matching** | **Final videos** |
| --- | --- | --- | --- | --- | --- |
| NCH / Training | 825 | 174 | 54 | N/A | 597 |
| OSU / Validation | 105 | 0 | 0 | N/A | 105 |
| VUMC / External paired test | 104 paired videos | 0 | 0 | 0 | 104 |

*Note. N/A indicates that the exclusion category was not applicable to that split. The OSU-validation and VUMC external paired test cohorts were not subjected to additional case-level exclusion after cohort assignment.*

### Complete per-class diagnostic performance

Complete one-versus-rest performance estimates for the CNN-only, tympanometry-only, and hybrid models are provided in Table B in S1 Text. These values complement the main-text hybrid-model summary by reporting all three methods with 95% confidence intervals.

Table B in S1 Text. Complete one-versus-rest per-class performance of CNN-only, tympanometry-only, and hybrid models with 95% confidence intervals.

| **Method** | **Class** | **n** | **Accuracy (95% CI)** | **Sensitivity (95% CI)** | **Specificity (95% CI)** | **Balanced accuracy (95% CI)** |
| --- | --- | --- | --- | --- | --- | --- |
| CNN-only | Effusion | 11 | 90.38 (83.20–94.69) | 36.36 (15.17–64.62) | 96.77 (90.94–98.90) | 66.57 (52.39–82.25) |
| CNN-only | Normal | 35 | 85.58 (77.56–91.06) | 97.14 (85.47–99.49) | 79.71 (68.78–87.51) | 88.43 (82.61–93.50) |
| CNN-only | Perforation | 20 | 78.85 (70.04–85.59) | 85.00 (63.96–94.76) | 77.38 (67.35–85.01) | 81.19 (71.27–89.76) |
| CNN-only | Retraction | 20 | 80.77 (72.15–87.19) | 0.00 (0.00–16.11) | 100.00 (95.63–100.00) | 50.00 (50.00–50.00) |
| CNN-only | Tympanostomy tube | 10 | 96.15 (90.53–98.49) | 70.00 (39.68–89.22) | 98.94 (94.22–99.81) | 84.47 (68.23–99.49) |
| CNN-only | Tympanosclerosis | 8 | 95.19 (89.24–97.93) | 50.00 (21.52–78.48) | 98.96 (94.33–99.82) | 74.48 (55.73–93.23) |
| Tympanometry-only | Effusion | 11 | 85.58 (77.56–91.06) | 54.55 (28.01–78.73) | 89.25 (81.33–94.05) | 71.90 (56.50–87.55) |
| Tympanometry-only | Normal | 35 | 75.00 (65.89–82.33) | 85.71 (70.62–93.74) | 69.57 (57.92–79.15) | 77.64 (69.27–85.38) |
| Tympanometry-only | Perforation | 20 | 88.46 (80.91–93.28) | 70.00 (48.10–85.45) | 92.86 (85.28–96.69) | 81.43 (70.45–91.47) |
| Tympanometry-only | Retraction | 20 | 77.88 (69.00–84.79) | 35.00 (18.12–56.71) | 88.10 (79.46–93.40) | 61.55 (50.41–73.07) |
| Tympanometry-only | Tympanostomy tube | 10 | 90.38 (83.20–94.69) | 0.00 (0.00–27.75) | 100.00 (96.07–100.00) | 50.00 (50.00–50.00) |
| Tympanometry-only | Tympanosclerosis | 8 | 92.31 (85.55–96.05) | 0.00 (0.00–32.44) | 100.00 (96.15–100.00) | 50.00 (50.00–50.00) |
| Hybrid | Effusion | 11 | 91.35 (84.37–95.38) | 72.73 (43.44–90.25) | 93.55 (86.63–97.01) | 83.14 (67.98–96.91) |
| Hybrid | Normal | 35 | 80.77 (72.15–87.19) | 94.29 (81.39–98.42) | 73.91 (62.49–82.81) | 84.10 (77.35–90.23) |
| Hybrid | Perforation | 20 | 94.23 (87.98–97.33) | 90.00 (69.90–97.21) | 95.24 (88.39–98.13) | 92.62 (84.78–98.80) |
| Hybrid | Retraction | 20 | 83.65 (75.37–89.54) | 60.00 (38.66–78.12) | 89.29 (80.88–94.26) | 74.64 (62.86–85.69) |
| Hybrid | Tympanostomy tube | 10 | 97.12 (91.86–99.01) | 80.00 (49.02–94.33) | 98.94 (94.22–99.81) | 89.47 (74.49–100.00) |
| Hybrid | Tympanosclerosis | 8 | 96.15 (90.53–98.49) | 75.00 (40.93–92.85) | 97.92 (92.72–99.43) | 86.46 (68.75–99.50) |

*Note. Values are percentages with 95% confidence intervals unless otherwise indicated. Accuracy, sensitivity, and specificity intervals were calculated using Wilson binomial intervals; balanced-accuracy intervals were estimated using bootstrap resampling.*

### Patient-level sensitivity analysis for bilateral-ear contribution

The external paired cohort included 104 ear-level examinations from 76 unique patients. Twenty-eight patients contributed bilateral ear examinations, corresponding to 56 ear-level examinations, whereas 48 patients contributed one ear examination. No examination had an unparsed ear side; the cohort included 56 right-ear and 48 left-ear examinations.

In the patient-clustered bootstrap analysis, the hybrid model achieved an overall accuracy of 81.73% (95% CI: 74.87-88.49), compared with 63.46% (95% CI: 56.46-71.97) for the CNN-only model and 54.81% (95% CI: 43.05-62.52) for tympanometry alone. The paired improvement of the hybrid model relative to the CNN-only model was 18.27 percentage points (95% CI: 9.61-27.59).

In the one-ear-per-patient sensitivity analysis, the hybrid model remained higher than the CNN-only model. Across 100 repeated random one-ear-per-patient selections, the mean overall accuracy was 82.64% for the hybrid model, 65.05% for the CNN-only model, and 56.05% for tympanometry alone. The mean paired improvement of the hybrid model relative to the CNN-only model was 17.59 percentage points. These analyses support that the observed performance improvement was not solely attributable to bilateral-ear contribution.

Table C in S1 Text. Patient and ear composition of the VUMC external paired cohort.

| **Measure** | **Value** |
| --- | --- |
| Ear-level examinations | 104 |
| Unique patients | 76 |
| Patients with bilateral ears | 28 |
| Patients with one ear | 48 |
| Ear examinations from bilateral-ear patients | 56 |
| Right ears | 56 |
| Left ears | 48 |
| Unparsed ear side | 0 |

Table D in S1 Text. Patient-clustered bootstrap analysis of overall diagnostic performance.

| **Metric** | **Method** | **Estimate (95% CI)** |
| --- | --- | --- |
| Overall accuracy | CNN Only | 63.46% (56.46–71.97) |
| Overall accuracy | Tympanometry Only | 54.81% (43.05–62.52) |
| Overall accuracy | Hybrid | 81.73% (74.87–88.49) |
| Paired accuracy improvement | Hybrid minus CNN | 18.27% (9.61–27.59) |

*Note. The patient-clustered bootstrap resampled patients with replacement and retained all available ear examinations from each sampled patient.*

Table E in S1 Text. One-ear-per-patient sensitivity analysis.

| **Metric** | **Method** | **Mean estimate (sensitivity range)** |
| --- | --- | --- |
| Overall accuracy | CNN Only | 65.05% (60.53–69.11) |
| Overall accuracy | Tympanometry Only | 56.05% (51.32–60.53) |
| Overall accuracy | Hybrid | 82.64% (78.95–85.53) |
| Paired accuracy improvement | Hybrid minus CNN | 17.59% (13.16–22.37) |

*Note. The interval represents the 2.5th-97.5th percentile across 100 repeated random selections of one ear per patient and should be interpreted as a sensitivity range rather than a formal confidence interval.*

### Systematic diagnostic disagreement between expert labels and model predictions

The CNN-only model demonstrated significant asymmetry in the multiclass contingency table comparing expert labels with model predictions. Bowker’s test was significant for the CNN-only model, with p=0.0052. The Stuart-Maxwell test also showed significant marginal inhomogeneity for the CNN-only model, with p=1.53 × 10⁻⁵. These results indicate that the CNN-only model exhibited systematic directional misclassification and that the distribution of CNN-only predictions differed significantly from the distribution of expert diagnostic labels.

In contrast, the hybrid model did not show significant asymmetry or marginal inhomogeneity. Bowker’s test for the hybrid model yielded p=0.753, and the Stuart-Maxwell test yielded p=0.309. These findings suggest that the hybrid model reduced directional diagnostic bias and produced a prediction distribution more consistent with expert labels.

Clinically, this indicates that adding tympanometry did not merely increase the number of correct predictions; it also changed the pattern of errors. The CNN-only model showed systematic diagnostic bias, whereas the hybrid model produced a more balanced error pattern across diagnostic categories.

Table F in S1 Text. Bowker’s test of symmetry and Stuart-Maxwell test of marginal homogeneity.

| Test | **CNN-only** | **Hybrid** |
| --- | --- | --- |
| Bowker’s test of symmetry, degrees of freedom | 15 | 15 |
| Bowker’s test of symmetry, p value | 0.0052 | 0.753 |
| Stuart-Maxwell test, degrees of freedom | 5 | 5 |
| Stuart-Maxwell test, p value | 1.53 × 10⁻⁵ | 0.309 |

*Note. Bowker’s test evaluates symmetry of off-diagonal misclassification patterns. The Stuart-Maxwell test evaluates marginal homogeneity between expert labels and model predictions.*

Table G in S1 Text. Expert labels versus CNN-only predictions.

| **Ground truth** | **Predicted effusion** | **Predicted normal** | **Predicted perforation** | **Predicted retraction** | **Predicted tube** | **Predicted tympanosclerosis** | **Row total** |
| --- | --- | --- | --- | --- | --- | --- | --- |
| Effusion | 4 | 5 | 2 | 0 | 0 | 0 | 11 |
| Normal | 0 | 34 | 1 | 0 | 0 | 0 | 35 |
| Perforation | 0 | 2 | 17 | 0 | 1 | 0 | 20 |
| Retraction | 3 | 6 | 10 | 0 | 0 | 1 | 20 |
| Tube | 0 | 1 | 2 | 0 | 7 | 0 | 10 |
| Tympanosclerosis | 0 | 0 | 4 | 0 | 0 | 4 | 8 |
| Column total | 7 | 48 | 36 | 0 | 8 | 5 | 104 |

*Note. Rows represent expert ground-truth labels, and columns represent model predictions.*

Table H in S1 Text. Expert labels versus hybrid model predictions.

| **Ground truth** | **Predicted effusion** | **Predicted normal** | **Predicted perforation** | **Predicted retraction** | **Predicted tube** | **Predicted tympanosclerosis** | **Row total** |
| --- | --- | --- | --- | --- | --- | --- | --- |
| Effusion | 8 | 3 | 0 | 0 | 0 | 0 | 11 |
| Normal | 0 | 33 | 1 | 1 | 0 | 0 | 35 |
| Perforation | 0 | 0 | 18 | 1 | 0 | 1 | 20 |
| Retraction | 0 | 5 | 2 | 12 | 0 | 1 | 20 |
| Tube | 1 | 1 | 0 | 0 | 8 | 0 | 10 |
| Tympanosclerosis | 1 | 0 | 0 | 1 | 0 | 6 | 8 |
| Column total | 10 | 42 | 21 | 15 | 8 | 8 | 104 |

*Note. Rows represent expert ground-truth labels, and columns represent model predictions.*

### One-versus-rest AUROC comparison between CNN-only and hybrid models

The hybrid model achieved higher one-versus-rest AUROC values than the CNN-only model across all six diagnostic categories. The largest improvement was observed for retraction, where AUROC increased from 0.50 with the CNN-only model to 0.78 with the hybrid model. This improvement remained statistically significant after Bonferroni correction, with DeLong p=7.8 × 10⁻⁷.

The hybrid model also showed higher AUROC values for effusion, increasing from 0.67 to 0.85, and perforation, increasing from 0.81 to 0.93. These comparisons reached nominal statistical significance but did not meet the Bonferroni-adjusted significance threshold. AUROC values were also numerically higher for normal ears, tympanostomy tubes, and tympanosclerosis, although these differences were not statistically significant.

Clinically, these findings suggest that tympanometry provides the greatest incremental diagnostic value for conditions where otoscopic appearance may overlap but middle-ear physiology differs, particularly retraction, effusion, and perforation.

Table I in S1 Text. One-versus-rest AUROC comparison between CNN-only and hybrid models.

| **Diagnosis** | **CNN-only AUROC** | **Hybrid AUROC** | **DeLong p value** | **95% CI for AUROC difference (Hybrid minus CNN-only)** |
| --- | --- | --- | --- | --- |
| Effusion | 0.67 | 0.85 | 0.015 | [0.0363, 0.3381] |
| Normal | 0.88 | 0.91 | 0.302 | [-0.0197, 0.0636] |
| Perforation | 0.81 | 0.93 | 0.049 | [0.0007, 0.2398] |
| Retraction | 0.50 | 0.78 | 7.8 × 10⁻⁷ | [0.1702, 0.3941] |
| Tympanostomy tube | 0.84 | 0.90 | 0.271 | [-0.0432, 0.1539] |
| Tympanosclerosis | 0.74 | 0.86 | 0.144 | [-0.0409, 0.2805] |

*Note. AUROC values were calculated using one-versus-rest analysis. P values were obtained using DeLong’s test for paired AUROC curves. Bonferroni correction across six diagnostic categories gives an adjusted significance threshold of p < 0.0083; only retraction remained significant after correction.*

## Supplementary Figures

Figure A in S1 Text. One-versus-rest ROC curves for CNN-only and hybrid models.
